# Impact of hospital lead extraction volume on management of cardiac implantable electronic device-associated infective endocarditis

**DOI:** 10.1093/europace/euae308

**Published:** 2024-12-27

**Authors:** Ari G Mandler, Christopher T Sciria, Edward V Kogan, Ilya Kim, Ilhwan Yeo, Matthew S Simon, Luke K Kim, James E Ip, Christopher F Liu, Steven M Markowitz, Bruce B Lerman, George Thomas, Jim W Cheung

**Affiliations:** Department of Medicine, Division of Cardiology, Weill Cornell Medicine—New York Presbyterian Hospital and Weill Cornell Cardiovascular Outcomes Research Group (CORG), 520 East 70th Street 4th Floor, New York, NY 10021, USA; Department of Medicine, Division of Cardiology, Weill Cornell Medicine—New York Presbyterian Hospital and Weill Cornell Cardiovascular Outcomes Research Group (CORG), 520 East 70th Street 4th Floor, New York, NY 10021, USA; Department of Medicine, Division of Cardiology, University of Rochester Medical Center, Rochester, NY, USA; Department of Medicine, Division of Cardiology, Weill Cornell Medicine—New York Presbyterian Hospital and Weill Cornell Cardiovascular Outcomes Research Group (CORG), 520 East 70th Street 4th Floor, New York, NY 10021, USA; Department of Medicine, Division of Cardiology, Weill Cornell Medicine—New York Presbyterian Hospital and Weill Cornell Cardiovascular Outcomes Research Group (CORG), 520 East 70th Street 4th Floor, New York, NY 10021, USA; Department of Medicine, Division of Cardiology, Weill Cornell Medicine—New York Presbyterian Hospital and Weill Cornell Cardiovascular Outcomes Research Group (CORG), 520 East 70th Street 4th Floor, New York, NY 10021, USA; Department of Medicine, Division of Infectious Diseases, Weill Cornell Medicine—New York Presbyterian Hospital, New York, NY, USA; Department of Medicine, Division of Cardiology, Weill Cornell Medicine—New York Presbyterian Hospital and Weill Cornell Cardiovascular Outcomes Research Group (CORG), 520 East 70th Street 4th Floor, New York, NY 10021, USA; Department of Medicine, Division of Cardiology, Weill Cornell Medicine—New York Presbyterian Hospital and Weill Cornell Cardiovascular Outcomes Research Group (CORG), 520 East 70th Street 4th Floor, New York, NY 10021, USA; Department of Medicine, Division of Cardiology, Weill Cornell Medicine—New York Presbyterian Hospital and Weill Cornell Cardiovascular Outcomes Research Group (CORG), 520 East 70th Street 4th Floor, New York, NY 10021, USA; Department of Medicine, Division of Cardiology, Weill Cornell Medicine—New York Presbyterian Hospital and Weill Cornell Cardiovascular Outcomes Research Group (CORG), 520 East 70th Street 4th Floor, New York, NY 10021, USA; Department of Medicine, Division of Cardiology, Weill Cornell Medicine—New York Presbyterian Hospital and Weill Cornell Cardiovascular Outcomes Research Group (CORG), 520 East 70th Street 4th Floor, New York, NY 10021, USA; Department of Medicine, Division of Cardiology, Weill Cornell Medicine—New York Presbyterian Hospital and Weill Cornell Cardiovascular Outcomes Research Group (CORG), 520 East 70th Street 4th Floor, New York, NY 10021, USA; Department of Medicine, Division of Cardiology, Weill Cornell Medicine—New York Presbyterian Hospital and Weill Cornell Cardiovascular Outcomes Research Group (CORG), 520 East 70th Street 4th Floor, New York, NY 10021, USA

**Keywords:** Cardiac implantable electronic device, Infective endocarditis, Transvenous lead extraction, Mortality

## Abstract

**Aims:**

Utilization of transvenous lead extraction/removal (TLE) for the management of cardiac implantable electronic device (CIED)-associated infective endocarditis (IE) remains low. The aim of this study was to examine the impact of hospital TLE procedural volume on TLE utilization and outcomes for patients with CIED-associated IE.

**Methods and results:**

Using the Nationwide Readmissions Database, we evaluated 21 545 admissions for patients (mean age 70 years, 39% female) with CIEDs hospitalized with IE at TLE centres. Hospitals were categorized based on annual volume tertiles: (i) low-volume (1–17 TLEs/year), (ii) medium-volume (18–45 TLEs/year), and (iii) high-volume centres (>45 TLEs/year). Between 2016 and 2019, 57% of admissions in the study were to low-volume TLE centres. Transvenous lead extraction/removal was performed during 6.9, 19.3, and 26% of admissions for CIED-associated IE at low-, medium-, and high-volume TLE centres, respectively (*P* < 0.001). After adjustment for age and comorbidities, hospitalization for IE at high-volume centres was independently associated with TLE when compared with low-volume centres (adjusted odds ratio 4.26; 95% confidence interval 3.53–5.15). Transvenous lead extraction/removal-associated complication rates were similar at 2.5, 2.3, and 3.4% at low-, medium-, and high-volume centres, respectively (*P* = 0.493). Overall inpatient mortality during admissions to low-, medium-, and high-volume centres was also similar.

**Conclusion:**

Admissions to high-volume TLE centres were associated with higher utilization of TLE for management of CIED-associated IE. Transvenous lead extraction/removal-associated complications and mortality among patients hospitalized with CIED-associated IE were similar when stratified by hospital TLE volume, but this needs to be considered in context of significant differences in patient comorbidity burden between centres.

## Graphical Abstract

**Graphical Abstract:**
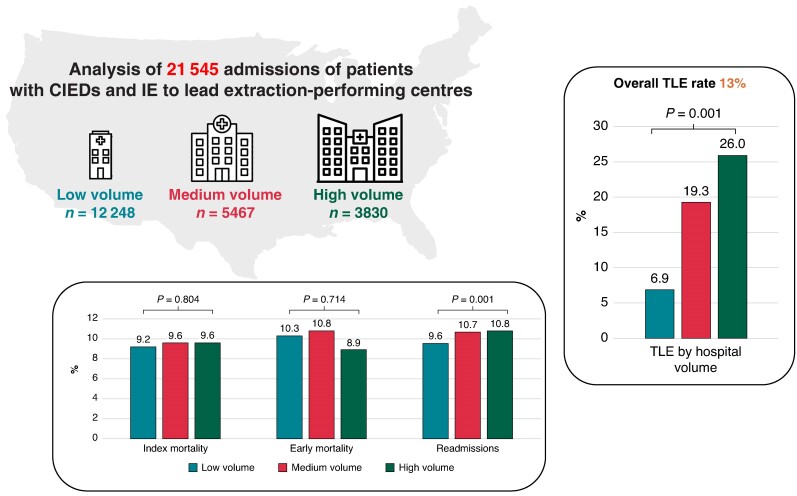
Impact of hospital TLE volume on TLE utilization for the management of CIED-associated endocarditis and its associated outcomes.

**What’s new?**

- The impact of institutional transvenous lead extraction (TLE)/removal volume for the management of cardiac implantable electronic device (CIED)-associated infective endocarditis (IE) has not been elucidated.
- In this study of over 21,000 admissions to TLE centres in the Nationwide Readmissions Database for CIED-associated IE, the proportion of patients undergoing TLE at low-volume TLE centres was as low as 7%.
- Across all centres stratified by TLE volume, TLE was associated with significantly lower in-hospital mortality.
- Transvenous lead extraction-associated complications and mortality among patients hospitalized with CIED-associated IE were similar when stratified by hospital TLE volume, likely due in part to significant differences in patient co-morbidity burden between centres.

## Introduction

Cardiac implantable electronic device (CIED)-related infections have increased over the past few decades, leading to significant morbidity, mortality, and healthcare costs.^1–3^ Current guidelines recommend complete CIED system removal for patients with infective endocarditis (IE), regardless of the presence of definite evidence of CIED involvement.^3–5^ Despite these recommendations, utilization of transvenous lead extraction/removal (TLE) in patients with CIEDs and IE in the United States remains low.^6^ Barriers to TLE utilization include physician lack of familiarity with established treatment guidelines and perceived risk of TLE-associated complications.^4,7,8^

Physician and hospital factors associated with increased experience with TLE will affect clinical judgment, operator technique, and access to equipment. The impact of hospital TLE volume on the management and outcomes of patients with CIED-related infections including IE has not been well studied. Therefore, using a nationally representative, all-payer administrative database, we sought to characterize hospital TLE volume between 2016 and 2019 and investigate the impact of hospital volume on TLE utilization among patients hospitalized with CIED-associated IE, TLE-associated complications, and overall 30-day readmissions and in-hospital mortality.

## Methods

### Study database

Data for this study were sourced from the Agency for Healthcare Research and Quality (AHRQ) Healthcare Cost and Utilization Project (HCUP)—Nationwide Readmissions Database (NRD) files spanning from 2016 to 19.^9,10^ The NRD serves as a repository of de-identified hospital inpatient discharges and readmissions allowing for national estimates of hospital utilization. It is an annual database from one calendar year of discharge data and utilizes verified patient linkage numbers to track hospital admissions within a state during a given year. Each entry in the NRD includes procedure and diagnosis codes according to the International Classification of Diseases, Tenth Revision, Clinical-Modification (ICD-10-CM) for every patient’s hospital discharge. Discharge records were weighted in accordance with the sampling scheme during these time periods, facilitating inferences for a nationally representative population. The study was deemed exempt by the Weill Cornell Medicine Institutional Review Board, as the HCUP–NRD is a publicly available database containing de-identified patient information. The data underlying this article will be shared on reasonable request to the corresponding author. The research reported in this paper adhered to the revised 2013 Helsinki Declaration guidelines.

### Study population

From January 2016 to November 2019, all hospitalizations for patients with CIEDs and IE were identified in the NRD. All patients had a pre-existing CIED, as identified by ICD-10-CM diagnosis codes for presence of cardiac pacemaker (Z95.0) or presence of implantable cardioverter–defibrillator (ICD) (Z95.810), in addition to ICD-10-CM codes for IE (B376, I330, I339, I38, and I39) (see Supplementary material online, *Table S1*). The exposure variable of TLE was identified using ICD-10-CM procedure codes (02PA0MZ, 02PA3MZ, and 02PA4MZ). Transvenous lead extraction/removal centres were identified by identifying all hospitals that performed at least one TLE procedure per year for the entire NRD between 2016 and 19. After excluding hospitalizations to a non-TLE centre, the study population comprised all hospitalizations for CIED-associated IE to a TLE centre. Because the NRD is reset annually, patients who were discharged in December from their index lead extraction were excluded from the study to ensure 30-day follow-up after discharge. Furthermore, patients younger than 18 years of age or those missing length of stay or mortality data were similarly excluded from the study.

### Clinical variables

Clinical variables at the patient level and hospital level were collected as baseline characteristics. Nationwide Readmissions Database variables were used to identify age, sex, income per zip code, and primary payer. Significant comorbidities and cardiac diagnoses included in the analysis were defined using ICD-10-CM codes or AHRQ comorbidity measures defined in Supplementary material online, *Table S2*. Hospital lead extraction volume was determined yearly using unique hospital identification numbers to calculate the total number of procedures performed by an institution each year. Because of the NRD’s design, hospitals were not monitored over the years. Consequently, the same hospital appearing in the NRD during different years was considered distinct. Hospitals were grouped into volume tertiles using annual procedural volume cut-offs based on 33rd and 67th percentiles of the total number of hospitalizations with TLE in the entire dataset between 2016 and 19 [low-volume tertile: 1–17 TLEs per year (excluding December); medium-volume tertile: 18–45 TLEs per year (excluding December); high-volume tertile: >45 TLEs per year (excluding December)].

### Study endpoints

The primary endpoint of this study was index admission mortality (defined as in-hospital mortality during index admission for IE in the presence of existing CIED) according to the methodology described by HCUP.^10,11^ Secondary endpoints included all-cause 30-day readmissions (only the first readmission within 30 days after discharge from index TLE), early mortality (defined as combined index and 30-day readmission mortality), and TLE-associated complications, which included: vascular complications (superior vena cava injury/repair, innominate vein injury/repair, and other vascular complications), open cardiac surgery, hematoma/haemorrhage, cardiac perforation/tamponade, and pneumothorax/hemothorax (see Supplementary material online, *Table S3*).

### Statistical analysis

Analyses were performed using SAS software, version 9.4 (SAS Institute, Cary, NC, USA). Survey-specific statements were utilized as per AHRQ recommendations (e.g. SURVEYFREQ, SURVEYMEANS, SURVEYLOGISTIC, and SURVEYREG). Discharge weight provided by the NRD was used to obtain national estimates.^9,10^ Categorical variables are shown as frequencies and continuous variables are presented as mean (SE), based on the normality of distribution. Baseline characteristics were compared by Rao–Scott χ^2^ test for categorical variables and either Mann–Whitney–Wilcoxon non-parametric test or survey-specific linear regression for continuous variables. To examine the independent association between TLE procedural volume and outcomes of interest, we created multivariable logistic regression models by including TLE volume tertiles and covariates that had univariate significance for each outcome (*P* < 0.10). All 95% confidence intervals (CI) and *P* values were corrected for multiple comparisons, and tests were two-sided with *P* values < 0.05 indicating statistical significance.

## Results

### Study population

**Figure 1:**
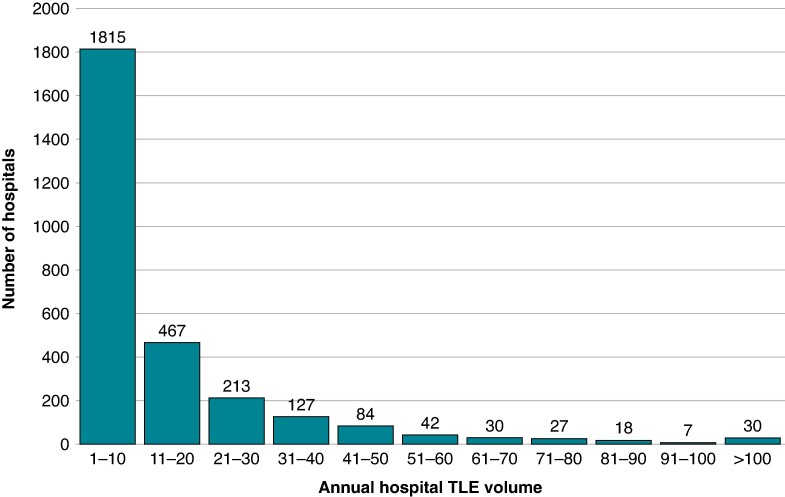
Distribution of annual TLE volume among TLE centres in the NRD between 2016 and 19. Lead extraction/removal procedures for the month of December were excluded. Identical hospitals across different years were treated as different hospitals due to the use of unique hospital identifiers in the NRD. Of 2860 hospitals, 1815 (63%) performed 10 or fewer TLE procedures per year. TLE, transvenous lead extraction/removal.

Between January 2016 and November 2019, there were 25 303 admission records for patients with CIEDs hospitalized for IE. After excluding 3758 admissions to non-TLE centres, the study cohort comprised 21 545 admissions for patients with CIED-associated IE to TLE centres. Overall, there were 12 248 (56.8%), 5467 (25.4%), and 3830 (17.8%) admissions to low-, medium-, and high-volume TLE centres, respectively. The distribution of overall annual TLE volume among TLE centres is depicted in *Figure 1*. There were 2179 (76%) low-volume tertile hospitals, 486 (17%) medium-volume tertile hospitals, and 195 (7%) high-volume tertile hospitals. There were significant differences in baseline characteristics between patients who presented to low-volume extraction centres as compared to those who presented to medium- and high-volume TLE centres (*Table 1*). Patients at low-volume TLE centres were more likely to be older and female and with a higher prevalence of chronic lung disease and dementia. Patients at high-volume TLE centres had more ICDs, obesity, congestive heart failure, cerebrovascular disease, chronic liver disease, coagulopathy, drug abuse, and *Staphylococcus aureus* infection compared with those at lower-volume TLE centres.

**Table 1:** Baseline characteristics of patients with CIEDs and IE stratified by hospital lead extraction volume

		Extraction centres			
Characteristics	Overall	Low-volume TLE	Medium-volume TLE	High-volume TLE	*P*-value
No. of admissions	21 545	12 248	5467	3830	
Age, mean (SE), year	70.3 (0.2)	72.3 (0.2)	69.0 (0.4)	66.0 (0.6)	<0.001
Age <65 years	6415 (29.8%)	3082 (25.2%)	1783 (32.6%)	1550 (40.5%)	
Age 65–74	4744 (22.0%)	2618 (21.4%)	1234 (22.6%)	892 (23.3%)	
Age ≥65 years	10 386 (48.2%)	6548 (53.5%)	2450 (44.8%)	1388 (36.2%)	
Female sex	8313 (38.6%)	4873 (39.8%)	2087 (38.2%)	1352 (35.3%)	0.003
Presence of ICD	7812 (36.3%)	3904 (31.9%)	2225 (40.7%)	1683 (43.9%)	<0.001
*Staphylococcus aureus* infection	4727 (21.9%)	2445 (20.0%)	1320 (24.2%)	962 (25.1%)	<0.001
Congestive heart failure	15 246 (70.8%)	8396 (68.6%)	3955 (72.4%)	2894 (75.6%)	<0.001
Hypertension	16 048 (74.5%)	9110 (74.4%)	4097 (74.9%)	2842 (74.2%)	0.817
Diabetes mellitus	8341 (38.7%)	4751 (38.8%)	2183 (39.9%)	1407 (36.7%)	0.118
Obesity	3748 (17.4%)	2020 (16.5%)	999 (18.3%)	729 (19.0%)	0.031
Cerebrovascular disease	2590 (12.0%)	1369 (11.2%)	728 (13.3%)	493 (12.9%)	0.02
Peripheral vascular disease	3161 (14.7%)	1842 (15.0%)	764 (14.0%)	555 (14.5%)	0.531
Chronic lung disease	6416 (30.0%)	3766 (30.8%)	1597 (29.2%)	1052 (27.5%)	0.040
Chronic liver disease	2288 (10.6%)	1209 (9.9%)	589 (10.8%)	490 (12.8%)	0.002
Coagulopathy	5192 (24.1%)	2695 (22.0%)	1436 (26.3%)	1061 (27.7%)	<0.001
Dementia	1985 (9.2%)	1284 (10.5%)	466 (8.5%)	235 (6.1%)	<0.001
Drug abuse	1446 (6.7%)	723 (5.9%)	399 (7.3%)	324 (8.5%)	0.003
Income per zip code					0.015
1st quartile (lowest)	6297 (29.6%)	3580 (29.6%)	1638 (30.3%)	1079 (28.5%)	
2nd quartile	6105 (28.7%)	3533 (29.2%)	1605 (29.7%)	968 (25.5%)	
3rd quartile	4786 (22.5%)	2711 (22.4%)	1232 (22.8%)	842 (22.2%)	
4th quartile (highest)	4096 (19.2%)	2268 (18.8%)	929 (17.2%)	899 (23.7%)	
Primary payer					<0.001
Medicare	16 345 (75.9%)	9686 (79.1%)	4019 (73.6%)	2640 (68.9%)	
Medicaid	1982 (9.2%)	1009 (8.2%)	505 (9.3%)	468 (12.2%)	
Private including HMO	2351 (10.9%)	1092 (8.9%)	690 (12.6%)	569 (14.9%)	
Self-pay/no charge/other	848 (3.9%)	452 (3.7%)	245 (4.5%)	152 (4.0%)	

HMO, health maintenance organization; ICD, implantable cardioverter–defibrillator; TLE, transvenous lead extraction/removal.

### Hospital transvenous lead extraction/removal volume and transvenous lead extraction/removal utilization for management of cardiac implantable electronic device-associated infective endocarditis

Transvenous lead extraction was performed in 13.5% of all study patients with utilization varying significantly across low-, medium-, and high-volume TLE centres. Transvenous lead extraction/removal occurred in 6.9% of hospital admissions to low-volume centres vs. 19.3 and 26.0% of hospital admissions to medium- and high-volume centres, respectively (*P* < 0.001). Multivariable analysis was performed to examine factors independently associated with TLE management. After adjustment for age, medical comorbidities, and demographic factors, patients with CIED-associated IE admitted to high-volume TLE centres were found to have significantly higher odds of management with TLE [adjusted odds ratio (aOR) 4.26 vs. low-volume TLE centres; 95% CI: (3.53–5.15); *P* < 0.001] (*Table 2*). Among patients with *S. aureus* infections, TLE was performed in 16.4, 36.6, and 41.4% of admissions to low-, medium-, and high-volume TLE centres, respectively (*P* < 0.001).

**Table 2:** Multivariable analysis of TLE management stratified by hospital lead extraction volume

	Adjusted odds ratio^a^	*P*-value
Low-volume TLE centres	Reference	Reference
Medium-volume TLE centres	2.96 (2.49–3.51)	<0.001
High-volume TLE centres	4.26 (3.53–5.15)	<0.001

^a^Adjusted for age, ICD placement, *S. aureu*s infection, gender, hypertension, diabetes mellitus, heart failure, obesity, kidney disease, cerebrovascular disease, coagulopathy, depression, drug use, and insurance type.

TLE, transvenous lead extraction/removal.

### Hospital TLE volume and TLE procedural complications

**Figure 2:**
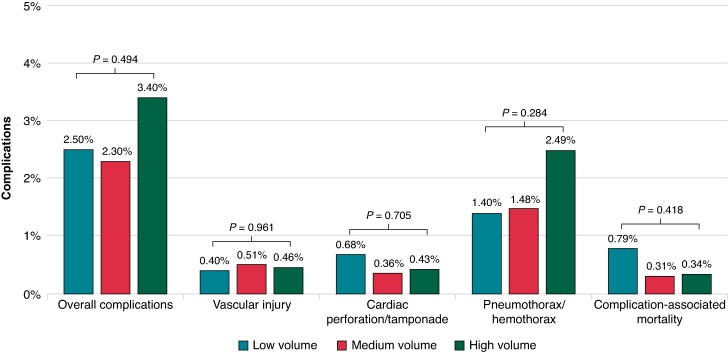
Unadjusted lead extraction-associated complications stratified by hospital volume. Complication-associated mortality was defined as presence of any TLE-associated complication and death during index hospitalization.

Among all patients (*n* = 2900) managed with TLE, procedural complications occurred in 2.5% of cases. Overall proportion of TLE-associated complications was not significantly different between low-, medium-, and high-volume TLE centres at 2.5, 2.3, and 3.4%, respectively (*P* = 0.494) (*Figure 2*). Specifically, the incidence of vascular complications (superior vena cava injury/repair, innominate vein injury/repair, and other vascular complications), cardiac perforation/tamponade, and pneumothorax/hemothorax were not significantly different across volume centres. In addition, TLE procedural complication-associated inpatient mortality was similar across low-, medium-, and high-volume TLE centres at 0.79, 0.31, and 0.34%, respectively (*P* = 0.418).

### Hospital transvenous lead extraction/removal volume and outcomes

**Figure 3:**
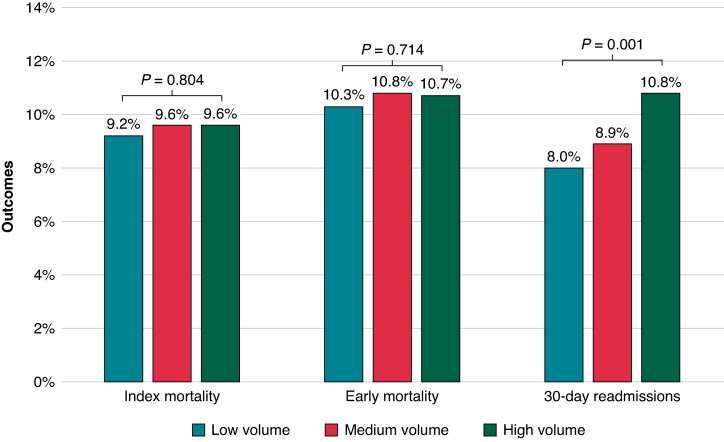
Unadjusted index mortality, early mortality, and 30-day readmissions stratified by hospital volume.

**Figure 4:**
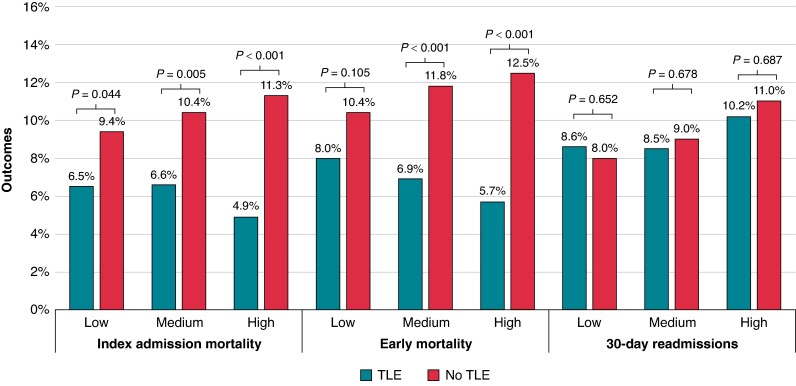
Comparison of outcomes among patients managed with or without lead extraction stratified by hospital volume. TLE, transvenous lead extraction/removal.

Overall, unadjusted rates of index mortality and early mortality during hospitalizations for CIED-associated IE were similar at low-, medium-, and high-volume TLE centres (*Figure 3*). Compared with low-volume TLE centres, 30-day readmissions were higher among patients admitted to high-volume TLE centres (8.0 vs. 10.8%; *P* = 0.001). Index mortality, early mortality, and 30-day readmissions as stratified by hospital TLE volume and presence or absence of TLE are summarized in *Figure 4*. Across each subgroup of patients at low-, medium-, and high-volume TLE centres, utilization of TLE was associated with significant lower index mortality. In addition, TLE management was associated with significantly lower early mortality at medium- and high-volume TLE centres. Utilization of TLE was not associated with lower 30-day readmissions across all TLE volume centres.

## Discussion

This study used a contemporary real-world, all-payer, nationally representative database to analyse over 21 500 admission records for patients with CIEDs and IE presenting to a lead extraction centre. In this study, we demonstrated several key findings (*Graphical Abstract*). First, overall utilization of TLE for management of patients with CIED-associated IE was low at 14%, with low-volume TLE centres having the lowest utilization at 7%. Secondly, there were significant differences in patient disease burden between those admitted to low-volume TLE centres and those admitted to high-volume TLE centres. Patients admitted to low-volume TLE centres were older and had more dementia, whereas those at high-volume TLE centres had a higher burden of cardiovascular disease, drug abuse, and *S. aureus* bacteraemia. Thirdly, overall rates of TLE-related complications were found to be low and not significantly different across volume centres, although this needs to be considered in the context of a higher comorbidity burden among patients admitted to high-volume TLE centres. Finally, overall mortality for patients admitted for CIED-associated IE was not found to be different across low-, medium-, and high-volume centres. Again, differences in comorbidity burden among patients treated at higher-volume centres compared with lower-volume centres likely contributed to this finding. This is the first study, to our knowledge, to examine the impact of overall institutional TLE volume on its utilization for the treatment of CIED infection.

### Transvenous lead extraction/removal utilization across extraction centres

There is growing evidence that real-world utilization of TLE for management of CIED-associated infections is low.^6^ Our study demonstrates that TLE utilization was lowest at low-volume TLE centres, where fewer than 1 in 14 patients with CIED-associated IE underwent TLE. There are likely several contributing factors for this observation. First, hospital-level factors including equipment access, nursing care quality, and availability of consulting services that are associated with institutional TLE volume will likely impact clinical decisioning to proceed with TLE. For example, increased access to and experience with mechanical and laser lead equipment may lower the threshold to proceed with complete lead removal in higher-risk cases with prolonged lead dwell times at high-volume centres.^11^ Similarly, the increased availability and expertise of cardiothoracic surgical back-up at high-volume centres may further influence decision-making for high-risk patients. Operator volume is also likely to play a role as seen in other cardiac procedures, but the NRD used in our study does not have provider-level data to allow for this analysis.^12,13^ Importantly, there were significant demographic and baseline characteristic differences between patients admitted to low- and high-volume TLE centres. Patients admitted to low-volume TLE centres were older and more likely to have dementia, which have previously been shown to be associated with lower TLE utilization.^6^ Concerns regarding increased risks of TLE in older patients with increased frailty or goals of care in patients with advanced dementia would likely lead to decisions not to perform TLE.

Knowledge gaps may also underlie low-volume TLE utilization. Differing levels of physician expertise with managing CIED infections between low- and high-volume TLE centres with respect to knowledge about recognition of CIED-associated infections and indications for CIED removal may have been present. A recent US-based survey found overall low physician familiarity with identifying and managing CIED infections.^7^ When questioned on the management of a pocket infection with methicillin-resistant *S. aureus* bacteraemia, only 65% of non-electrophysiology (EP) cardiologists and 33% of primary care physicians (PCPs) recommended complete system extraction despite the presence of a class I indication for complete system extraction in this scenario.^3^ Similar findings have also been seen in Europe-based survey studies, suggesting that major gaps in physician knowledge and skills related to CIED care are widespread.^8,14^

### Transvenous lead extraction/removal complications and outcomes across extraction centres

Perceived risks of TLE can deter referrals for the procedure. In a recent physician-based survey, 86% of respondents cited concerns about lead extraction as a major factor influencing their decision to refer patients for the procedure.^7^ Up to one-fourth of both non-EP cardiologists and PCPs perceive the risk of major complications associated with lead extraction to be high (6–10%) or very high (>10%). In our study, overall complication rates associated with TLE were markedly lower than these perceived figures at 2.45%, which are comparable to rates reported by prior registry studies.^15–18^ The GermAn Laser Lead Extraction RegistrY (GALLERY) showed a high clinical success rate (97.9%) and a low major complication rate (2.1%) among laser lead extractions across 24 German centres.^18^ Our study compared TLE complications and outcomes among centres across a wide range of procedural volume, including those performing fewer than 20 TLE procedures per year. Across different volume centres, there were no significant differences observed in the rates of overall TLE-associated complications or specific complications such as vascular injury and cardiac perforation or tamponade. Specifically, 0.45% of patients who underwent TLE experienced both a major TLE-associated complication and died, with no significant variation across volume centres. The European Lead Extraction ConTRolled Registry (ELECTRa) analysed TLE safety and efficacy across low- and high-volume extraction centres (low categorized as facilities that perform 1–29 TLEs/year and high categorized as facilities that perform ≥30 TLEs/year).^15^ Like our study, ELECTRa found no significant differences in major complications or procedure-related deaths between low- and high-volume centres. However, it did show higher rates of minor complications and early death within 30 days at lower-volume centres, which was also noted in a recent systematic review on the topic.^19^ Differences in study population (i.e. all patients undergoing lead extraction vs. patients with CIED-associated endocarditis) may have accounted for some of the differences in findings between our study and others. The absence of differences in TLE-related adverse events in our cohort among patients undergoing TLE at low-volume centres compared to those at high-volume centres was likely driven by significant differences in comorbidity burden. Patients treated at high-volume centres had more heart failure, cerebrovascular disease, livers disease, coagulopathy, and *S. aureus* infection. Additionally, we would emphasize that due to the limitations of the NRD, we were unable to examine lead dwell time, device type, and other patient factors as variables in our analysis. Physicians at lower-volume TLE centres may have been much less likely to perform higher-risk TLE cases involving patients with complex anatomy, longer lead dwell times, and devices with multiple leads. Moreover, patients deemed to be at higher risk of TLE procedures would be more likely to be referred or transferred to high-volume TLE centres by non-TLE-performing physicians. Regardless, we found that most TLE centres across the United States, as in Europe, performed fewer than 30 TLE procedures/year despite practice recommendations.^20,21^ In the context of current expert consensus statements on lead extraction that emphasize operator competency, more data are needed to establish institutional thresholds linked to improved patient outcomes.^22^

### Study limitations

There are several limitations to this study. First, this is a retrospective analysis based on data acquired from the NRD. The NRD only covers ∼50% of all hospitals in the United States and in 22 states, which may affect the generalizability of the findings.^9,10^ Secondly, the study relies on ICD-10-CM coding, which may pose challenges via missing or inappropriate coding although quality control measures attempt to mitigate this issue.^9,10^ Thirdly, while the NRD provides information about total hospital extraction volume, it does not provide operator-level data which prevents inclusion of operator volume as a variable. Fourthly, ICD-10-CM codes can be limited with respect to specificity. For example, the coding for presence of a cardiac pacemaker and automatic implantable cardiac defibrillator (Z95.0 and Z95.810, respectively) does not distinguish between transvenous and leadless pacemakers or subcutaneous ICDs. Therefore, a subset of patients included in this study do not have transvenous leads available for extraction. Furthermore, we could not gather data on the number of leads present in the CIED system. In addition, TLE-associated complications could have been over-estimated in our study as the use of ICD-10-CM codes in the NRD to define complications does not permit assignment of causality of each diagnosis to the procedures performed. Specifically, we did not explore the subgroup of patients who might have had concurrent valve surgery during index hospitalization which could have accounted for some of the complications that were recorded. Fifthly, specific clinical variables that could influence the decision to pursue TLE and patient outcomes, such as echocardiographic data, medication use, and procedural details (e.g. time to extraction from initial diagnosis, lead type and fixation, type, and number of extraction tools), are not captured in the NRD and therefore cannot be accounted for in multivariable analyses. Specifically, lead dwell times are not available in the NRD and it has been established that leads with longer dwell times are typically managed by high-volume centres.^23^ This factor could have impacted complication rates in this study. Therefore, conclusions with regard to complications and mortality associated with TLE across volume centres should be interpreted with caution. Sixthly, we did not collect data on concomitant valve surgeries that might have been performed during index hospitalization for CIED-associated IE which would have impacted complication and mortality outcomes. In general, we were unable to rule out the possibility that non-TLE procedures performed during the hospitalization could have accounted for some of the adverse events recorded. Lastly, mortality data only track during hospitalizations and therefore out-of-hospital deaths are not included, which would affect the reported early mortality data in this study.

## Conclusions

Hospital transvenous lead extraction/removal procedural volume is significantly associated with utilization of TLE in the management of CIED-associated IE. In this study, TLE complication rates, including associated major complications, were low and not significantly different across volume extraction centres, although this was likely impacted by significantly higher comorbidity burden among patients admitted to high-volume centres compared with low-volume centres. Patients who presented to high-volume centres vs. low-volume centres were significantly more likely to undergo lead extraction. This is despite the higher cardiovascular comorbidity burden among patients admitted to high-volume TLE centres compared with low-volume centres. Across all volume centres, TLE management was associated with significantly lower all-cause mortality. Improved access to TLE for patients with CIEDs and systemic infections is warranted and may lead to improved outcomes.

## Supplementary Material

euae308_Supplementary_Data

## Data Availability

The data underlying this article will be shared on reasonable request to the corresponding author. Tweet: Hospital lead extraction volume impacts the management of patients with CIED-related infections. What can be done to limit disparities in care? #CIEDinfection.
