# Supplementary material for: Impact of hospital lead extraction volume on management of cardiac implantable electronic device-associated infective endocarditis
Source: Europace. 2024 Dec 27;27(1):euae308. doi: 10.1093/europace/euae308 (PMC11707385; doi:10.1093/europace/euae308)
Supplement: euae308_Supplementary_Data [file euae308_supplementary_data.docx]

**SUPPLEMENTAL MATERIALS**

**Supplemental Table 1:** Inclusion and Exclusion Criteria for Selection of Study Discharge Records

| **Inclusion Criteria** | **ICD-10 Codes** |
| --- | --- |
| **Cardiac Implantable Electronic Device** |  |
| Presence of cardiac pacemaker | Z95.0 |
| Presence of automatic (implantable) cardiac defibrillator | Z95.810 |
| **Endocarditis** |  |
| Candidal endocarditis | B376 |
| Acute and subacute infective endocarditis | I33.0 |
| Acute and subacute endocarditis, unspecified | I33.9 |
| Endocarditis, valve unspecified | I38 |
| Endocarditis and heart valve disorders in disease classified elsewhere | I39 |
| **Staph Aureus Infections** |  |
| Sepsis due to Staphylococcus aureus | A410 |
| Sepsis due to Methicillin susceptible Staphylococcus aureus | A4101 |
| Sepsis due to Methicillin resistant Staphylococcus aureus | A4102 |
| Methicillin susceptible Staphylococcus aureus infection, unspecified site | A4901 |
| Methicillin resistant Staphylococcus aureus infection, unspecified site | A4902 |
| Staphylococcus aureus as a cause of diseases classified elsewhere | B956 |
| Methicillin susceptible Staphylococcus aureus infection as the cause of diseases classified elsewhere | B9561 |
| Methicillin resistant Staphylococcus aureus infection as the cause of diseases classified elsewhere | B9562 |
| Pneumonia due to Methicillin susceptible Staphylococcus aureus | J15211 |
| Pneumonia due to Methicillin resistant Staphylococcus aureus | J15212 |
| **Transvenous Lead Extraction** |  |
| Removal of Cardiac Lead from Heart, Open Approach | 02PA0MZ |
| Removal of Cardiac Lead from Heart, Percutaneous Approach | 02PA3MZ |
| Removal of Cardiac Lead from Heart, Percutaneous Endoscopic Approach | 02PA4MZ |

ICD-10-CM = International Classification of Diseases, Tenth Edition, Clinical Modification

**Supplemental Table 2:** ICD-10-CM, CCS and AHRQ NRD Codes Used to Define Comorbidities at Index Admission

| **Comorbidities** | **NRD Elixhauser Codes** |
| --- | --- |
| **Hypertension** | HTN_CX, HTN_UNCX |
| **Diabetes Mellitus** | DIAB_CX, DIAB_UNCX |
| **Human Immunodeficiency Virus (HIV)** | AIDS |
| **Alcohol Use Disorder** | ALCOHOL |
| **Drug Abuse** | DRUG_ABUSE |
| **Anemia** | ANEMDF, BLDLOSS |
| **Leukemia/Lymphoma** | CANCER_LEUK, CANCER_LYMPH |
| **Metastatic Cancer** | CANCER_METS |
| **Solid Tumor Malignancy without Metastasis** | CANCER_SOLID, CANCER_NSITU |
| **Cerebrovascular Disease** | CBVD |
| **Heart Failure** | CHF |
| **Coagulopathy** | COAG |
| **Dementia** | DEMENTIA |
| **Depression** | DEPRESS |
| **Liver Disease** | LIVER_MLD, LIVER_SEV |
| **Chronic Pulmonary Disease** | LUNG_CHRONIC |
| **Neurological Disorders** | NEURO_MOVT, NEURO_OTH, NEURO_SEIZ |
| **Obesity** | OBESE |
| **Peripheral Vascular Disease** | PERIVASC |
| **Kidney Disease** | RENLFL_MOD, RENLFL_SEV |

NRD = Nationwide Readmissions Database

**Supplemental Table 3:** ICD-10-CM, CCS and AHRQ NRD Codes Used to Define Complications at Index Admission

| **Comorbidities** | **ICD-10 Codes** |
| --- | --- |
| **Cardiac Tamponade/Cardiac Perforation** | Cardiac Tamponade (I31.4)  Hemopericardium (I31.2)  Drainage of the pericardial cavity, with drainage device, open approach (0W9D00Z)  Drainage of the pericardial cavity, open approach, diagnostic (0W9D0ZX)  Drainage of the pericardial cavity, open approach (0W9D0ZZ)  Drainage of Pericardial Cavity with Drainage Device, Percutaneous Endoscopic Approach (0W9D40Z) Drainage of Pericardial Cavity, Percutaneous Endoscopic Approach (0W9D4ZX)  Drainage of Pericardial Cavity, Percutaneous Endoscopic Approach (0W9D4ZZ) |
| **Vascular complications** | **SVC repair**  Repair Superior Vena Cava, Open Approach (02QV0ZZ)  Repair Superior Vena Cava, Percutaneous Approach (02QV3ZZ)  Repair Superior Vena Cava, Percutaneous Endoscopic Approach (02QV4ZZ)  Unspecified Injury of the SVC - initial encounter (S2520XA)  **Innominate vein repair**  Repair Right Innominate Vein, Open Approach (05Q30ZZ)  Repair Right Innominate Vein, Percutaneous Approach (05Q33ZZ)  Repair Right Innominate Vein, Percutaneous Endoscopic Approach (05Q34ZZ)  Repair Left Innominate Vein, Open Approach (05Q40ZZ)  Repair Left Innominate Vein, Percutaneous Approach (05Q43ZZ)  Repair Left Innominate Vein, Percutaneous Endoscopic Approach (05Q44ZZ)  **Other Vascular Complications**  Unspecified injury of unspecified blood vessel of thorax, initial encounter (S2590XA)  Accidental puncture and laceration of a circulatory system organ or structure during a circulatory system procedure (I9751)  accidental puncture and laceration of a circulatory system organ or structure during other procedure (I9752)  Complication of mesenteric artery following a procedure, not elsewhere classified, initial encounter (T81710A)  Complication of renal artery following a procedure, not elsewhere classified, initial encounter (T81711A)  Complication of other artery following a procedure, not elsewhere classified, initial encounter (T81718A)  Complication of vein following a procedure, not elsewhere classified, initial encounter (T8172XA) |
| **Hematoma or hemorrhage** | Intraoperative hemorrhage and hematoma of a circulatory system organ or structure complicating a cardiac catheterization (I97410)  Intraoperative hemorrhage and hematoma of a circulatory system organ or structure complicating other circulatory system procedure (I97418)  Intraoperative hemorrhage and hematoma of a circulatory system organ or structure complicating other procedure (I9742)  Postprocedural hemorrhage and hematoma of a circulatory system organ or structure following a cardiac catheterization (I97610)  Postprocedural hemorrhage and hematoma of a circulatory system organ or structure following other circulatory system procedure (I97618)  Postprocedural hemorrhage and hematoma of a circulatory system organ or structure following other procedure (I9762)  Hemorrhage due to cardiac prosthetic devices, implants and grafts, initial encounter (T82837A)  hemorrhage of vascular prosthetic devices, implants and grafts, initial encounter (T82838A) |
| **Hemothorax/Pneumothorax** | Postprocedural Pneumothorax (J95.811)  Hemothorax (J94.2)  Traumatic pneumothorax, initial encounter (S270XXA)  Traumatic hemothorax, initial encounter (S271XXA)  Traumatic hemopneumothorax, initial encounter (S272XXA)  Other pneumothorax (J9383)  Pneumothorax, unspecified (J939)  Drainage of Right Pleural Cavity with Drainage Device, Percutaneous Approach (0W9930Z)  Drainage of Left Pleural Cavity with Drainage Device, Percutaneous Approach (0W9B30Z) |
| **Open Cardiac Surgery** | Repair Heart, Open Approach (02QA0ZZ)  Repair Heart, Percutaneous Approach (02QA3ZZ)  Repair Right Heart, Open Approach (02QB0ZZ)  Repair Heart, Percutaneous Endoscopic Approach (02QA4ZZ)  Repair Right Heart, Percutaneous Approach (02QB3ZZ)  Repair Left Heart, Open Approach (02QC0ZZ)  Repair Left Heart, Percutaneous Approach (02QC3ZZ)  Repair Right Heart, Percutaneous Endoscopic Approach (02QB4ZZ)  Repair left heart, percutaneous endoscopic approach (02QC4ZZ) |
